# Supplementary material for: Uncovering the Role of PdePrx12 Peroxidase in Enhancing Disease Resistance in Poplar Trees
Source: J Fungi (Basel). 2023 Mar 27;9(4):410. doi: 10.3390/jof9040410 (PMC10142663; doi:10.3390/jof9040410)
Supplement: Supplementary file 1 [file jof-09-00410-s001.zip › 10.jof-2232238-supplementary/supplementary file/Data S1.pdf]

DNA, coding DNA sequence (CDS) and protein sequences of PdePRX12.

>PdePrx12_DNA_3114 bp
aaagactcaagaaactaaccaaaccatggccagagctgctatgtccttcacctctcttcttctgatttcttctctctttctagcttcttggttttgtgcc
actgaagcacaaggcaccatccctatagtgaatggcctgtcatggacattctacaagtctaaatgccctaaagttgaatccattataaggaaaca
gctggagaaggtgttcaagaaggatatcgaacaagctgctggcttgcttcgtctccacttccatgattgctttgttcaggttacaggatatttcatat
attcaaaactcatcttggcatttactattatgttcagttgcaaggttcactatttttgatcaaagaattaatgtctaaaatttaactctgcagggatgtga
tggttcagtattgcttgatggatcggcgagtggtccaagcgagcaggacgcccctcccaacctgaccttgagagcaaaggcgtttgagatcat
cgaagacctacgccggcgagtggaaaaggcttgcggccttgtcgtctcctgttctgatattcttgcgcttgcagcccgtgactctgtttacctggt
gagtcgacttgaatcactctctgttatcgtagtttttgataatgatcctagctagccagtatggagcacatgatttaataccatctcctgtatcgttag
atacacatccgtacatatatagcccagccatattcttgagagtcgagtctaaatcgagtttttttttttttgaaaaatagctttttccatatatattttttt
aaaaagtagtgtacttgattttttttcaaaaaatagcacacttaataaattaatttgaaaacagtggtaattaattatctttctttaggaaatataggaat
gagatttgggttcattgattttaataatacaattaaaaattagcctaattatggctcttgttagatgttaagtaacttatttttataacataaaaataatata
ttgagaaattaaacgttcagtataaagcttagatttttaaaataaaattgaaatttaattatccaaatttattatggtatttctggaataagtgtaaaataa
ctaaatacacatgaaataattacaaatagaagattacgagaaagtcaagtctttctgtccccatataatatatagtcctgcaacaaggggcataaa
atgtgactgatcagaccagataagcttatccaatgttatcttcagagcttagatctccgcctgtcgatttctttccagttagttgatcagtgattgcga
gaggcaaattaattcctcataatcttttaagagcccatcatttcatattatggttactagcgattgtttatttaaatattttttatttaaaaatatattaaagt
aatattttttttagatttattttgactttaatattaatatgttaaaataatctaaaaatactaaaaaaaaatttatttttctcaaaaatactcttacccagcaacat
tattattatcaccttcttgtgacttgtcatgatcattaaacacatataaagggtgtatatatgcatggctccttgagttctgctttgttaaaaaaaatatttt
ttattttcaaattaagttttagatttttttatgttgatattaatttttttttattctttttttaaaaaaacattttgtatcacattccccgtgtgtgtaataaatcaa
ataaaatttctttcggtaacaagtcattactcataaccaaaagctaatcctttttaacttctatgcattttgtctactgcagtctggtggcccagattata
atgttcccttgggaaggcgagacggtctaaaatttgcaactcaaaatgaaaccttagacaacctcccgccaccccctttgctaacgccgacacaat
tctctcatctctcgccaccaagggcttggatgccaccgacgtggtagccctgtccggtggccacaccattggtataagtcactgcagctctttca
ctgaccgcctctatccaacccaagatcctacaatggacaaaacctttgccaacaatctcaaggaagtttgccccacaagagacttcaataacac
aaccgtgttggatattcgatctcctaataaatttgacaacaagtactatgttgatctcatgaatcgccaaggcctgtttacttcagaccaggacttgt
acacgaacaagaagacaaggggcattgtcactagctttgctgttaatcaaagtttgttctttgataagtttgtggttgcaatgatcaaaatgtcaca
gctcaaggtcttgacaggaaatcaaggtgaaatccgcgcgccagttgctcggagagaaattcaggctacagttacttggagtctgtggtggaaga
gggtctcgacgcactgtccggattaatatgaaagaagtaaattctaatacatgaaaggccaaattccactagctagataaaaaatgctgggtc
gttgtgcacttgttaattatgggttcgttgctgtcttttttttttaacaaaatgctgatgctatcttcttctgacaacttcaattctgcttgttcttaataaaat
agtcgaggatgtaacatatgagtttggatggctaataggacgttggctttgtcaaagttttataatttataattacggtttgttctagactaaagttga
gatttttgttcctggcgattagccctctcccattgctctctgtgtcaaggctaagcaaatcccaaatgggcatgcgttttagttttccaaatagggct
gtctatggcccatcgatccatgtcacaaatttttagttacattcctggattggaccatctttgatgcgctttgatcaagaactttccacgagagtagct
aaagaatttaggtttggttatggatttgtttttcaaaatttatcagtaaatctcacgagttttgaaaaattaattgagggatgtgtcagttaatccaaac
acttactattaacaaaaaattataatttttttttaatgcatcttaagaatcaaatttaaaagggactaaaactgtgcaaacattcaattttccttataaaca
ttcaattttccttttttaatgctcaacctccctat

>PdePrx12_CDS_1065 bp
atggccagagctgctatgtccttcacctctcttcttctgatttcttctctctttctagcttcttggttttgtgccactgaagcacaaggcaccatccctat
agtgaatggcctgtcatggacattctacaagtctaaatgccctaaagttgaatccattataaggaaacagctggagaaggtgttcaagaaggat
atcgaacaagctgctggcttgcttcgtctccacttccatgattgctttgttcagggatgtgatggttcagtattgcttgatggatcggcgagtggtc
caagcgagcaggacgcccctcccaacctgaccttgagagcaaaggcgtttgagatcatcgaagacctacgccggcgagtggaaaaggctt
gcggccttgtcgtctcctgttctgatattcttgcgcttgcagcccgtgactctgtttacctgtctggtggcccagattataatgttcccttgggaagg
cgagacggtctaaaatttgcaactcaaaatgaaaccttagacaacctcccgccaccccctttgctaacgccgacacaattctctcatctctcgccac
caagggcttggatgccaccgacgtggtagccctgtccggtggccacaccattggtataagtcactgcagctctttcactgaccgcctctatcca

acccaagatcctacaatggacaaaacctttgccaacaatctcaaggaagtttgccccacaagagacttcaataacacaaccgtgttggatattc
gatctcctaataaatttgacaacaagtactatgttgatctcatgaatcgccaaggcctgtttacttcagaccaggacttgtacacgaacaagaaga
caaggggcattgtcactagctttgctgttaatcaaagtttgttctttgataagtttgtggttgcaatgatcaaaatgtcacagctcaaggtcttgaca
ggaaatcaaggtgaaatccgcgccagttgctcggagagaaattcaggctacagttacttggagtctgtggtggaagagggtctcgacgcact
gtccggattaatatga

>PdePrx12_protein_354 AA
MARAAMSFTSLLLISSLFLASWFCATEAQGTIPIVNGLSWTFYKSKCPKVESIIRKQLEKVF
KKDIEQAAGLLRLHFHDCFVQGCDGSVLLDGSASGPSEQDAPPNLTLRAKAFEIIEDLRRR
VEKACGLVVSCSDILALAARDSVYLSGGPDYNVPLGRRDGLKFATQNETLDNLPPPFANA
DTILSSLATKGLDATDVVALSGGHTIGISHCSSFTDRLYPTQDPTMDKTFANNLKEVCPTRD
FNNTTVLDIRSPNKFDNKYYVDLMNRQGLFTSDQDLYTNKKTRGIVTSFAVNQSLFFDKF
VVAMIKMSQLKVLTGNQGEIRASCSERNSGYSYLESVVEEGLDALSGLI
